# Combined Structural and Functional 3D Plant Imaging Using Structure from Motion

**DOI:** 10.3390/s25051572

**Published:** 2025-03-04

**Authors:** Alim Yolalmaz, Jos de Wit, Jeroen Kalkman

**Affiliations:** Department of Imaging Physics, TU Delft, Lorentzweg 1, 2628 CJ Delft, The Netherlandsj.dewit-1@tudelft.nl (J.d.W.)

**Keywords:** structure from motion, computer vision, plant imaging, 3D imaging

## Abstract

We show non-invasive 3D plant disease imaging using automated monocular vision-based structure from motion. We optimize the number of key points in an image pair by using a small angular step size and detection in the extra green channel. Furthermore, we upsample the images to increase the number of key points. With the same setup, we obtain functional fluorescence information that we map onto the 3D structural plant image, in this way obtaining a combined functional and 3D structural plant image using a single setup.

## 1. Introduction

3D imaging is an important field that is used in a variety of application fields such as process control [1], cultural heritage [2], bioimaging [3], and archaeology [4]. In contrast to 2D imaging, 3D imaging eliminates occlusions, provides depth/height information of an object, and records the full structural information of the object. In the field of agriculture, 3D imaging plays an increasingly important role as it is an essential tool for monitoring plant growth from the cellular level up to the level of entire crop fields.

There are various non-destructive techniques to image the 3D structure of plants such as optical coherence tomography (OCT), which has been used for imaging Arabidopsis and measuring leaf thickness [5] and plant infection [6], light detection and ranging (LIDAR) which has been used for visualizing whole plants [7], canopies [8] and trees [9]. In addition to the aforementioned active techniques, passive techniques exist, like monocular-vision applied to sunflower and soybean [10], binocular-vision for imaging pachira glabra [11] and soybean plants [12], structure from motion (SfM), which was applied to cotton [13] and eggplant [14] and sugar beet [15]. Moreover, fusions of active and passive methods show great performance, such as demonstrated by a combination of LIDAR and SfM applied to vegetation structure [16], a fusion of LIDAR, SfM, and a microbolometer sensor for measuring Gossypium species structure [17], and utilization of SfM and simultaneous multi-view stereovision for Ocimum basilicum [18] and nursery paprika [19].

Most of these techniques, like LIDAR and multi-sensor techniques, are costly and/or involve complicated multi-modality data analysis. Most importantly, they only provide structural information and not functional information of the plant. Functional information is important because it gives more information about the actual state of the plant. Functional information can be obtained from the spectral data, which is useful as the color of the plant gives information about the processes that occur in the plant such as senescence [20], dehydration [21]. Fluorescence imaging provides information on photosynthesis [22], and metabolic responses to stress [23], and plant infection processes [24,25,26]. However, combining structural information with functional information commonly uses an additional imaging setup.

SfM is a 3D reconstruction technique that provides a 3D image of an object while preserving the spectral information of the object [27]. Depth information in SfM is obtained from the relative motion of point-like features in two images acquired at slightly different angles [28]. SfM has been applied for the reconstruction of dynamic scenes of non-rigid objects [29], height estimation of sorghum plants [30], and imaging of wheat crops [31].

In this study, we present the implementation of a monocular vision-based SfM technique to 3D visualize the combination of plant structure and disease in a lettuce plant using structural and UV-induced blue-green fluorescence image data, respectively. The setup only uses a single monochrome camera and a rotation stage to reconstruct the 3D structure of a lettuce pot plant. To capture more image features and create a highly detailed image of the surface texture, we optimize the image contrast and number of identified key points. In this way we provide a cost-effective method using a single imaging system to provide both structural and functional plant information.

## 2. Methods

### 2.1. Experimental Setup

**Figure 1:**
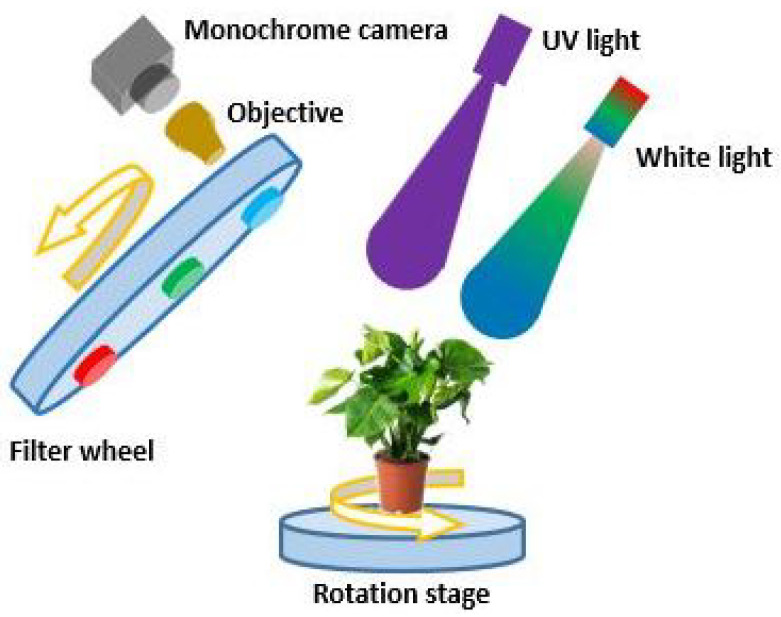
Experimental setup for structural and functional SfM 3D plant imaging. The setup consists of a white light source, a UV light source, a rotation stage, one filter wheel, a demagnifying objective, and a monochrome camera.

A schematic of the experimental setup is shown in Figure 1. The setup consists of a white-light source (LDL2-33X8SW, CCS), a UV light source (LDL-138X12UV2-365, CCS), a monochrome camera (acA1440-220um, Basler), an objective lens (C125-0818-5M-P f: 8 mm, Basler), three spectral filters (BP635, BP525 and BP470, Midwest Optical Systems Inc.) that transmit light in red, green, and blue color mounted on a filter wheel (FW102, Thorlabs). Infected areas of lettuce leaves show an enhanced fluorescence intensity in the blue-green spectrum between 400 nm and 560 nm [26] (ch.4). The blue and green filters were chosen to cover this area of enhanced emission and sufficiently overlap with the green and blue channel of normal RGB imaging. The blue fluorescence of the veins made the green channel most selective as biomarker for infection. The red filter was chosen on the lower wavelength side to avoid camera saturation due to the high fluorescence at the red edge of the visible light spectrum [26] (ch.4). The sample is mounted on a rotation stage (PRMTZ8/M, Thorlabs). The monochrome camera has 1440 × 1080 pixels, square pixels with a size of 3.45 µm, and a frame rate of 227 fps. To demagnify the plant and fit its entire image on the camera, the objective with a focal length of 8 mm is mounted on the monochrome camera providing a pixel resolution of 120 µm. The angular field of view (FoV) of the objective is 58-by-45 degrees. The distance between the objective and the monochrome camera is adjusted to create a sharp image of the lettuce pot plant. The orientation and distance of the camera is set to visualize the whole plant with minimal occlusion.

The white light source illuminates the lettuce cultivar Salinas plant to acquire a structural image of the plant. Red, green, and blue spectral filters can be rotated in front of the camera are used to sequentially collect images of the plant at different spectral bands. After obtaining structural images using the RGB filters, the UV light source illuminates the plant to attain UV-induced fluorescent images using the RGB spectral filters, which are chosen to optimize the blue-green fluorescence that is indicative of infection [24,26]. Subsequently, the rotation stage changes the perspective of the plant after obtaining structural and fluorescence images. The filter wheel, light sources, monochrome camera, and rotation stage are controlled by a script run in Python 3.11.5. For our setup, the acquisition speed is limited by the rate of filter wheel rotation.

### 2.2. Camera Calibration and Image Processing

The imaging setup was computationally calibrated to correct for optical distortions presented in all plant images. To obtain the calibration parameters, images of a 7 × 9 squares checkerboard calibration sample with 13 mm square size were collected from various orientations. The MATLAB estimateCameraParameters function was used to compute a homography, expressing the projective transformation between the world points and the image points, to obtain the calibration parameters. From the correspondences of multiple images of the checkerboard calibration sample the camera parameters were obtained. The calibration procedure also provided intrinsic parameters such as the focal length of the objective *f* and the principal points ($u_{0}$, $v_{0}$) expressing the center point of the camera that are necessary to reconstruct the 3D image of the plant, as defined in Equation (1). Using $k_{u}$ and $k_{v}$, which are the number of pixels per mm on the *x* and *y* axes, we obtained the intrinsic matrix of the camera K as presented in Equation (1).

(1) $$K=\left(\begin{array}{cccc} fk_{u} & 0 & u_{0} & 0\\ 0 & fk_{v} & v_{0} & 0\\ 0 & 0 & 1 & 0 \end{array}\right).$$

3D image reconstruction starts after acquiring an R, G, and B image of a lettuce plant from two different perspectives. The images are captured in a sealed enclosure to control environmental parameters such as temperature, airflow, and ambient illumination. To process the images, they are converted into five different spectral channels: red, green, blue, grayscale, and extra-green. The grayscale images are computed by averaging the intensity values at each pixel in the red, green, and blue channels. The ExG images are determined by employing the extra-green (ExG) algorithm through

(2) ExG(x,y)=2×I(x,y,G)−I(x,y,R)−I(x,y,B).

where I(x,y,G), I(x,y,R), and I(x,y,B) represent the pixel values of the images obtained with the green, red, and blue filters, respectively.

### 2.3. 3D Image Reconstruction

In SfM depth information is obtained from two images after locating the shift of a key point in two images acquired at different angles. A key point ${\tilde{X}}_{j}$ is a feature located in an image frame *j* that resembles a part of the structure. To identify the key points, first, the RGB images were converted into ExG images. Then, the images were digitally upsampled using cubic interpolation before all key points in each image were detected by employing the scale-invariant feature transform (SIFT) algorithm [32]. The SIFT algorithm identified each key point and labeled its location within a certain diameter. Therefore, we can only obtain depth information within the diameter of a key point. Then the key points in an image pair are matched with the Fast Library for Approximate Nearest Neighbors (FLANN) matcher [33]. The FLANN-based matcher coupled two key points in two images by considering parameters as the distance ratio of 0.6, FLANN index kdtree of 1, and trees of 5.

A key point describing a part of a structure in the world coordinate system is expressed as $\left[\begin{array}{ccc} X & Y & Z \end{array}\right]$. These coordinates need to be recovered SfM from a subsequent measurement of the same point on the pixel coordinate $\left[\begin{array}{cc} u & v \end{array}\right]$. A homogeneous 3D coordinate of the key point has the form $\left[\begin{array}{cccc} \tilde{X} & \tilde{Y} & \tilde{Z} & \tilde{W} \end{array}\right]$ with a nonzero scale factor $\tilde{W}$. The relation between the homogeneous 3D coordinate and the pixel coordinate is based on a 4×4 perspective transformation matrix Q according to

(3) $${\left[\begin{array}{cccc} \tilde{X} & \tilde{Y} & \tilde{Z} & \tilde{W} \end{array}\right]}^{⊤}=Q{\left[\begin{array}{cccc} u & v & 1/d(u,v) & 1 \end{array}\right]}^{⊤}.$$

The disparity d(u,v) in Equation (3) is the difference in the location of the same key points in two images. Using the disparity and mathematical and geometric relations, the homogeneous 3D coordinates of a disparity point are estimated with Equation (3). Here, the matrix Q is

(4) $$Q=\left(\begin{array}{cccc} 1 & 0 & 0 & -u_{0}\\ 0 & -1 & 0 & v_{0}\\ 0 & 0 & sfk_{u} & 0\\ 0 & 0 & 0 & 1 \end{array}\right).$$

The perspective transformation matrix Q is obtained from the camera calibration and bears details of the camera intrinsic matrix as seen in Equation (4) where the parameter s=0.02 is obtained with our calibration. Lastly, we express the coordinates of a key point in the world coordinates by dividing the homogeneous 3D coordinates by $\tilde{W}$ as $\left[\begin{array}{ccc} X & Y & Z \end{array}\right]$ = $\left[\begin{array}{ccc} \tilde{X}/\tilde{W} & \tilde{Y}/\tilde{W} & \tilde{Z}/\tilde{W} \end{array}\right]$. The data processing time for generating a 3D image is one minute using two images on a desktop PC (Intel(R) Xeon(R) CPU E5-1660 v3 @ 3.00 GHz).

## 3. Results

To make a good 3D plant image acquisition, we investigate important data acquisition and processing parameters of the imaging system such as the number of camera pixels, the angle step size between a consecutive image pair, and the spectral information of the plant. Most importantly, we increase the number of matched key points to create a high-resolution 3D reconstruction.

**Figure 2:**
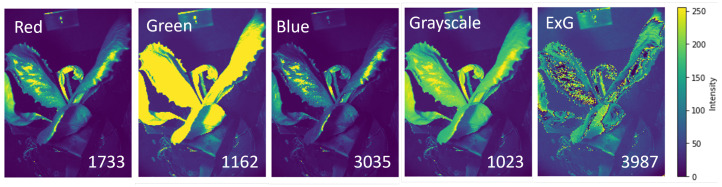
The image of the pot plant under different spectral filters with the number of matched key points indicated for 1 degree angle step.

First, we investigate the effect of the spectral information on the number of matched key points. The SIFT key point detection approach identifies key points in a complex image based on the presence of contrast. The contrast is strongly affected by the functional properties of the plant, the plant geometry, the scattering of light over the plant, the signal intensity, and the noise. In Figure 2, we demonstrate the intensity variation of one plant image in different spectral channels. The numbers of matched key points for each spectral image are as follows: 1733 for red, 1162 for green, 3035 for blue, 1023 for grayscale, and 3987 for ExG. The ExG image yields the highest number of matched key points, and, as a result, generates high contrast variation over the plant. As seen here, due to the intensity saturation in the image under the green filter, we encounter less number of matched key points compared to red and blue color channels because of the presence of smooth intensity variation over the plant in the green channel. Further 3D reconstruction is performed with the ExG data.

Second, we investigate increasing the number of key points. The number of pixels in the monochrome camera is 1.5 million (1080-by-1440), imposing a limit on the number of key points and features in each image. To monitor the pot plant with a higher number of matched key points, each plant image is digitally upsampled using cubic interpolation to a higher imaging format of 5400-by-7200 pixels. As a result of this operation, the number of matched key points for the images varies from 1856 for 1.5 million pixels to 3987 in the case of 38.9 million pixels. The upsampling increases the computational cost. When we upsample images from the imaging format of 1080-by-1440 pixels (1.4 s) to 3240-by-4320 pixels and to the maximum of 5400-by-7200 pixels, the calculation duration becomes 14 times (20 s) and 41 times (60 s) longer compared to the case without the upscaling on the same PC, respectively. However, this is still affordable with an average PC. The digital upsampling enables the detection of a higher numbers of matched key points in the images, which provides a more accurate 3D image formation. For further results, we use digitally upsampled images containing 38.9 million pixels.

**Figure 3:**
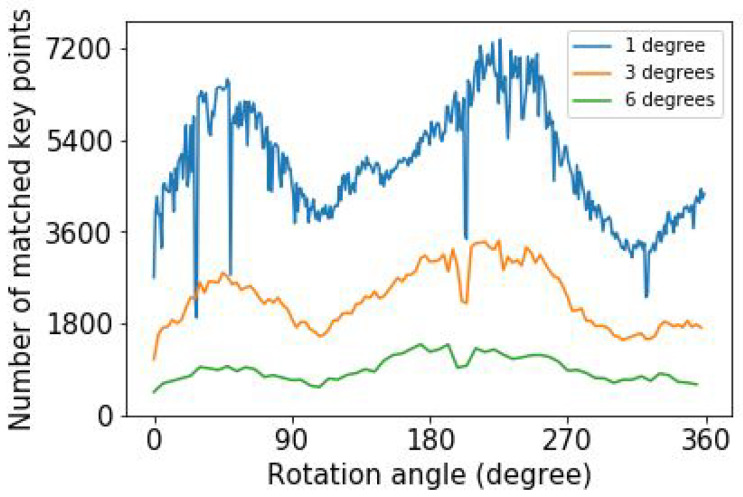
The number of matched key points in ExG plant image pairs as a function of rotation angle for three different angle step sizes for maximum upsampled images.

Third, we investigate how the number of matched key points depends on the angle step size. The number of matched key points differs for the various image pairs because of the variation of the structure in each image pair. Figure 3 shows the variation of the number of matched key points as a function of rotation angle for different angular step sizes for maximum upsampled images. When the angle difference between two consecutive images increases, the number of matched key points decreases, as seen in Figure 3. In general, there is a variation in the number of key points due to the variation of the plant structure as a function of angle.

**Figure 4:**
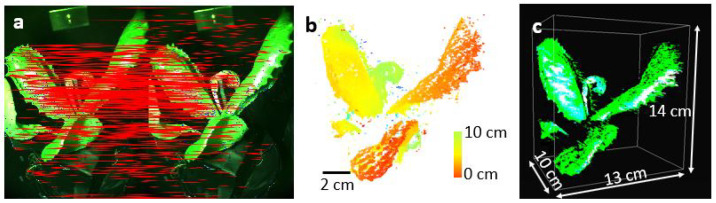
(**a**) The RGB images of the pot plant obtained under white light illumination at 1 degree and 2 degrees, respectively. The key points in the image pair are connected by red lines. (**b**) The 3D image of the plant with varying colors indicates depth variation. The color transition from red points to green points demonstrates the nearest and the farther points in the plant with respect to the camera location, respectively. (**c**) The 3D-rendered image of the plant with 7.2 million points is visualized in 3D coordinates.

Figure 4a shows two 2D ExG images of the pot plant attained at a 1-degree angle difference under white light illumination obtained from three images obtained with the RGB color filters. The images from the two perspectives dominantly carry the same view of the plant in addition to a small new structure that is occluded. Thanks to the overlapping structure occupying similar key points, successfully, 3987 matched key points in the image pair were detected and used to reconstruct the surface of the leaves. Based on the key point perspective change, the depth of the key points from the camera plane is reconstructed, as shown in Figure 4b. Figure 4c shows the rendered image of the plant structure in 3D, with overlaid the ExG intensity. Based on the calibrated camera magnification, and the pixel size of the camera, the 3D structure of the plant is scaled to obtain the plant structure coordinates in the world coordinate frame. The plant is visualized along three axes with respect, as can be seen in Figure 4c.

**Figure 5:**
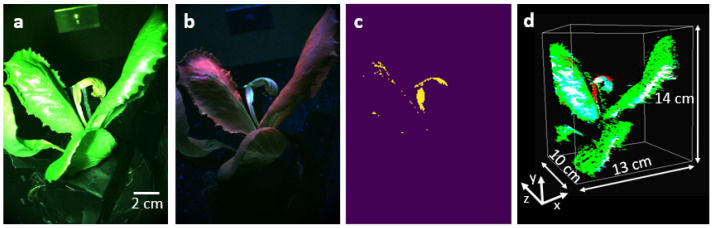
The RGB image of the plant under (**a**) the illumination of the white light source, (**b**) the illumination of the UV light source. (**c**) The plant disease points in the green channel of the image in (**b**) after performing the thresholding operation. (**d**) The 3D image of the plant using structural and fluorescence signals. The red points demonstrate disease signals.

Finally, we capture functional information of the lettuce pot plant infected with Bremia lactucae race BI:33EU, as shown in Figure 5. Figure 5a shows the RGB image of the plant under white light illumination, no infection is visible. The same plant under the UV light illumination visualized with the RGB spectral filters is shown in Figure 5b. The UV light source emits radiation at a spectral window centered around 365 nm, and this spectral region is not sensed by the monochrome camera positioned after the RGB filters. Thus, during this data collection operation with UV light on, the camera only monitors fluorescence signals emanating from the lettuce plant. The image of the lettuce pot plant in the green channel under UV illumination corresponds to fluorescence signals. We filter these points from weaker non-specific autofluorescence by tuning the thresholding parameter based on visual verification to preserve only signals related to the infected area corresponding to the plant disease. The thresholding parameter removes signals smaller than 110 counts to keep only the plant disease signals. As seen in Figure 5c, one of the leaves contains a high green fluorescent signal, which is related to the presence of plant disease [24,26]. In this image, there are 285771 disease pixels on the surface of the plant. They are mainly located in the infected area, but the plant veins also carry some similar signals.

Lastly, we register the UV fluorescence disease signal onto the 3D image of the plant, shown in Figure 4c. The generation of a 3D image based on UV-image key points is not possible due to the low number of features in the green image of the plant under UV illumination. Therefore, we created a 2D color map demonstrating the disease points in red color, and then registered the color map onto the 3D rendered image of the plant as seen in Figure 5d. The non-red signals in the color map demonstrate the healthy parts of the plant. Considering the number of structure points and the number of disease points, about 4% of the plant surface is covered with disease.

## 4. Discussion and Conclusions

We have demonstrated the 3D visualization of the structure and function of the lettuce plant using a single camera, acquisition at two angles, and only 7 images (2 RGB images under white-light illumination and 1 green image in fluorescence). Our results demonstrate a high-fidelity representation of the plant surface. Thanks to different data processing techniques, we increase the number of key points in an image pair to improve the accuracy of the plant structure in 3D. Moreover, we demonstrate the effect of the image processing techniques on the number of key points that lead to improving the feature detection which is the most important issue in the field of photogrammetry. Compared to other 3D imaging techniques like the monocular-vision and LIDAR, our method has more spectral information, has an enhanced number of key points, and is realized in a cost-effective instrument.

Successfull 3D plant reconstruction is based on obtaining a sufficient number of keypoints. This can be obtained by optimizing the light source intensity, camera integration time, and optical properties of the spectral filters. The lettuce plant turned out to have a sufficient number of key points in the ExG channel, with many features visible in the red and blue channels. For application to other plants and lighting conditions, the combination of color channels can be optimized to achieve a similar number of key points. An automated procedure for optimization of the data collection parameters can be implemented to produce consistent output for different plants and/or environmental conditions.

This work can be further developed by implementing artificial neural network for image segmentation aimed at plant disease identification [26,34,35,36,37]. Moreover, different color segmentation approaches such as HSV color space (H: hue, S: saturation, V: color brightness value) may enable the identification of more and better plant features [12]. This, for example, can be applied to estimate chlorophyll content for vegetation growth status, assess plant damage, and determine plant aging of eggplants [14].

Our method can aid in the detection of plant disease, its spatial distribution over the plant, and progression over time. This information is of critical for the fundamental study of plant-pathogen interactions. In addition, it can of significant aid in the (automated) plant phenotyping, which is pivotal for breeding better and more resistant crops.

## Data Availability

The code and data sets for reproducing the results are available in 4TU repository at https://doi.org/10.4121/e6db8707-10ee-4553-9a98-753f1b4c526a.
